# A Genome-Wide Screen for Machinery Involved in Downregulation of MHC Class I by HIV-1 Nef

**DOI:** 10.1371/journal.pone.0140404

**Published:** 2015-10-14

**Authors:** Maja K. Choma, Jennifer Lumb, Patrycja Kozik, Margaret S. Robinson

**Affiliations:** University of Cambridge, Cambridge Institute for Medical Research, Cambridge, CB2 0XY, United Kingdom; Helmholtz Zentrum Muenchen—German Research Center for Environmental Health, GERMANY

## Abstract

The HIV-1-encoded protein, Nef, plays a key role in the development of AIDS. One of Nef’s functions is to keep MHC class I off the surface of infected cells, a process that requires the host proteins clathrin and AP-1. To identify other proteins involved in this pathway, we carried out a genome-wide siRNA library screen on HeLa cells co-expressing HLA-A2 and an inducible form of Nef. Out of 21,121 siRNA pools, 100 were selected for further analysis, based on their ability to either inhibit or enhance downregulation of MHC-I by Nef. When cells were treated with the same siRNA pools as those used in the screen, 79% produced a similar phenotype. However, when the cells were treated with different siRNA reagents targeting the same genes, only 16% produced a similar phenotype. This indicates that most of the hits found in the original screen are likely to have been off-target, an important concern that is often not taken into account in siRNA screening studies. Nevertheless, we identified novel host factors involved in Nef-induced downregulation of MHC-I, including four genes, MIIP, CAMSAP3, SLC6A3, and KCTD19, where multiple reagents produced a strong inhibitory effect on Nef activity. Other hits slightly below our very high stringency cutoff point may also deserve further study. Thus, our dataset is a valuable resource for scientists investigating the pathogenesis of HIV.

## Introduction

Human Immunodeficiency Virus (HIV) is a major health concern, with an estimated 34 million people infected worldwide in 2013 (World Health Organisation). Unless treated with antiretroviral drugs, most HIV-positive individuals go on to develop Acquired Immune Deficiency Syndrome (AIDS), and the death toll from HIV/AIDS-related illnesses in 2013 alone was assessed at 1.5 million. However, there are rare instances (0.3%) of long-term non-progressors, who are able to control their HIV infection without medication. These individuals include the Sydney Blood Bank Cohort: eight people who were infected in the early 1980s with a strain of HIV-1 lacking the gene for Nef. Even after 30 years, three members of the Sydney Blood Bank Cohort still showed no signs of illness, highlighting the importance of Nef in the progression from HIV infection to full-blown AIDS [1,2].

Nef is an N-terminally myrsitoylated protein of approximately 27 kD, which has several functions, including downregulation of MHC class I (MHC-I) from the cell surface [3]. MHC-I downregulation by Nef is a highly conserved activity of primate lentiviruses, which correlates with protection from CTL lysis in vitro and in vivo [4]. Many other viruses have also adopted strategies to evade the immune system of the host, using mechanisms ranging from ER-associated degradation to endocytosis [5]. In the case of HIV-1 Nef, RNAi knockdown studies have shown that the adaptor protein complex 1 (AP-1) and clathrin are required for efficient downregulation of MHC-I [6,7]. Insights into the molecular mechanism of Nef-induced MHC-I downregulation were obtained from the crystal structure of a tripartite complex, consisting of Nef, the cytoplasmic tail of MHC-I, and the medium subunit of AP-1 (μ1). The structure shows that Nef is able to bind to both MHC-I and AP-1, and thus link the two together [8].

The current view is that Nef converts MHC-I into an AP-1-dependent clathrin-coated vesicle (CCV) cargo protein, which causes it to be diverted away from the constitutive secretory pathway and trafficked from the TGN to endosomes. Then, instead of cycling back and forth between the TGN and endosomes like many other AP-1-dependent cargo proteins [9,10], MHC-I goes on to be degraded in lysosomes, presumably by making use of other cellular machinery. Intriguingly, when clathrin-mediated endocytosis is inhibited by knocking down the plasma membrane-associated AP complex, AP-2, Nef is actually more effective at downregulating MHC-I [7]. It is not clear why this occurs, but it may be an indirect effect of the knockdown: e.g., there may be changes in the dynamics of MHC-I-containing endosomes. Thus, although AP-1 and clathrin are clearly important players in Nef-mediated downregulation of MHC-I, there must be other players as well, which have yet to be identified.

One way of finding such players would be to carry out a genome-wide siRNA library screen for Nef activity. Such screens have been carried out in the past to identify host proteins essential for successful HIV infection. Using libraries of ~20,000 different siRNAs, Brass et al. [11]identified and validated 276 hits that affected HIV replication; Zhou et al. [12]identified 232 hits that blocked viral entry and infectivity; and König et al. [13]found 213 hits inhibiting infectivity. Most of these hits were either novel proteins or proteins acting in pathways not previously implicated in HIV replication. However, the overlap between the hits of the three screens was small, which might be due to technical differences in how the screens were performed and analysed. The three screens used different strains of HIV, different host cell lines, and different siRNA libraries. They also employed different screen metrics and different hit criteria. In addition, there are many different steps in HIV infection, and effects on any one of these steps could produce a phenotype. Therefore, we hoped that by investigating a more narrow phenotype—i.e., downregulation of MHC-I by Nef—and by applying stringent analysis and validation criteria, we could find more reproducible hits. Furthermore, hits from our screen could be targets for drugs designed to interfere with immunoevasion rather than infection, emulating the phenotype of some of the long-term non-progressors.

**Fig 1:**
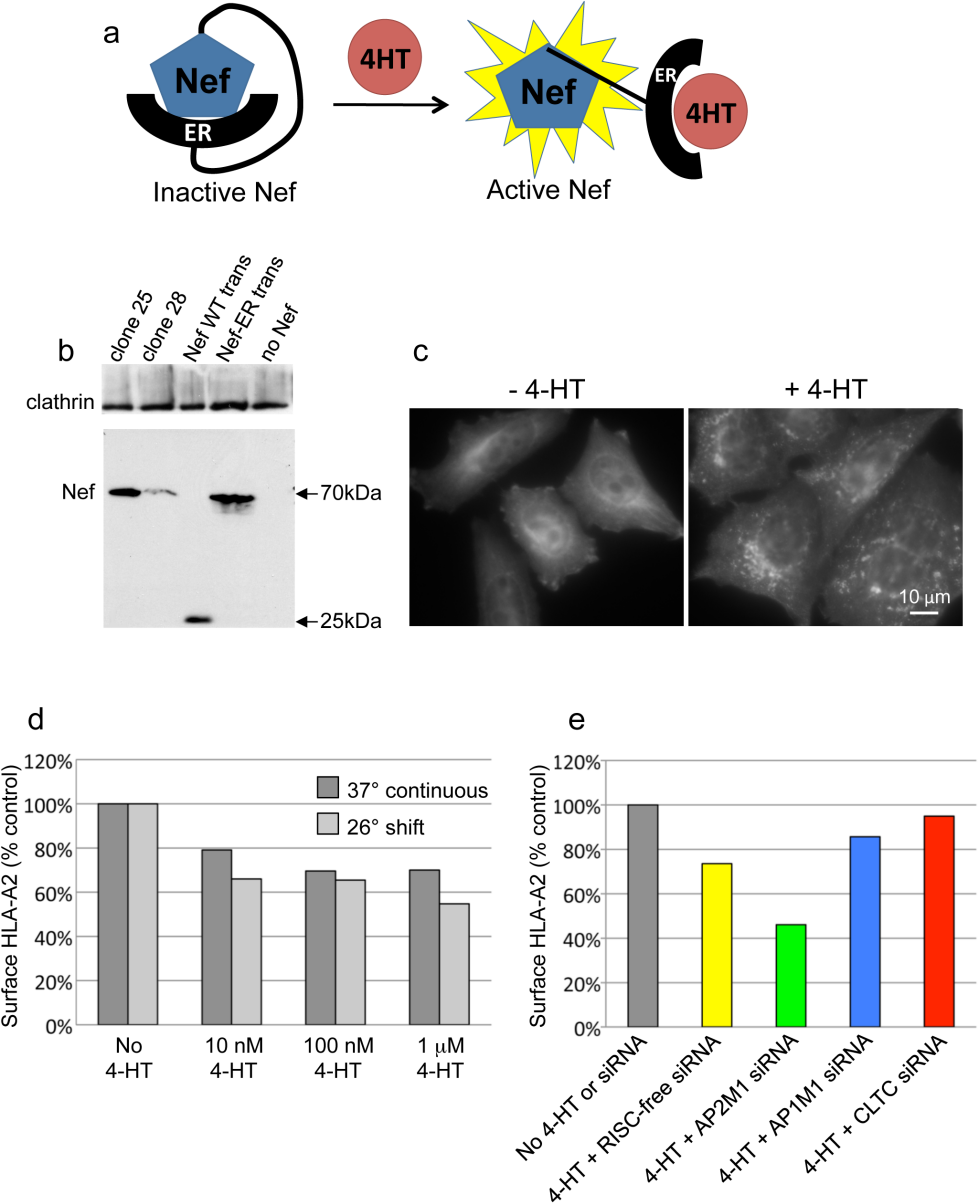
Establishing an inducible Nef system in HeLa cells. a, Schematic representation of the Nef-ER construct and its mode of action. b, Western blot of stably and transiently transfected cells, labelled with anti-Nef and with anti-clathrin as a loading control. Clone 25 and clone 28 are two different cell lines stably expressing Nef-ER. The cells in the Nef WT trans and Nef-ER trans lanes were transiently transfected with wild-type Nef and with Nef-ER, respectively. c, Cells stably expressing Nef-ER were either left untreated or treated with 100 nM 4HT for 48 hours, then labelled with an antibody against Nef. The label is diffuse and cytosolic in the absence of the drug, but becomes membrane-associated after the drug is added. d, Surface HLA-A2 in cells treated with different concentrations of 4HT for 48 hours, either continuously at 37°C or with a shift to 26°C after 24 hours. e, Surface HLA-A2 in 4-HT-treated and control cells, with or without various siRNA knockdowns.

Our lab previously carried out a genome-wide siRNA library screen to monitor changes in the surface expression of two different constructs with well-characterised clathrin-dependent sorting signals in their cytoplasmic tails [14]. Here we adopted a similar approach to investigate MHC-I downregulation. We carried out our screen in HeLa cells, because they are particularly amenable to siRNA knockdowns, and we used a stable cell line that expresses HLA-A2, which is much more susceptible to Nef than endogenous HeLa MHC-I [7]. In addition, in order to avoid the toxic effects of long-term Nef expression, we stably transduced the cells with a chimeric construct consisting of full-length Nef fused to the hormone-binding domain of the estrogen receptor (Nef-ER) [15]. In the absence of an ER agonist, the Nef is inactive, but addition of 4-hydroxytamoxifen (4-HT) induces Nef activity (Fig 1A).

## Results

### Establishing an assay system

Nef-ER was introduced into HLA-A2-expressing HeLa cells using a lentiviral system. Single cell clones were selected and tested first by Western blotting. Previous studies have shown that Nef activity is highly dose-dependent [7,16], so we were aiming for expression levels at least as high as those obtained by transient transfection. Fig 1B shows that Nef expression varied between clones, but we identified one cell line, clone 25, with comparable expression to cells that had been transiently transfected with either wild-type Nef or Nef-ER. We also assessed our clones by immunofluorescence, and found that in the absence of 4-HT, Nef-ER had a diffuse cytosolic distribution, but that addition of 4-HT caused the construct to localise to membranes, particularly in the juxtanuclear region of the cell (Fig 1C).

Next, we examined the effects of different concentrations of 4-HT on downregulation of HLA-A2 (Fig 1D). Cells were incubated with the drug for 48 hours, either continuously at 37°C, or with a shift to 26°C for the second half of the incubation, which has been shown to enhance Nef activity in HeLa cells [7,17]. Although we never saw more than a 50% loss of surface HLA-A2, most likely because the Nef-ER construct was expressed at only moderate levels, we found an increase in downregulation when cells were shifted to 26°C.

Using 100 nM 4-HT and a 26°C shift, we went on to test the effects of different siRNA knockdowns (Fig 1E). In agreement with previous reports, knocking down clathrin (CLTC) or the medium subunit of AP-1 (AP1M1) blocked Nef activity [7,18], while knocking down the medium subunit of AP-2 (AP2M1) enhanced Nef activity [7] [7].

### Genome-wide screen

**Fig 2:**
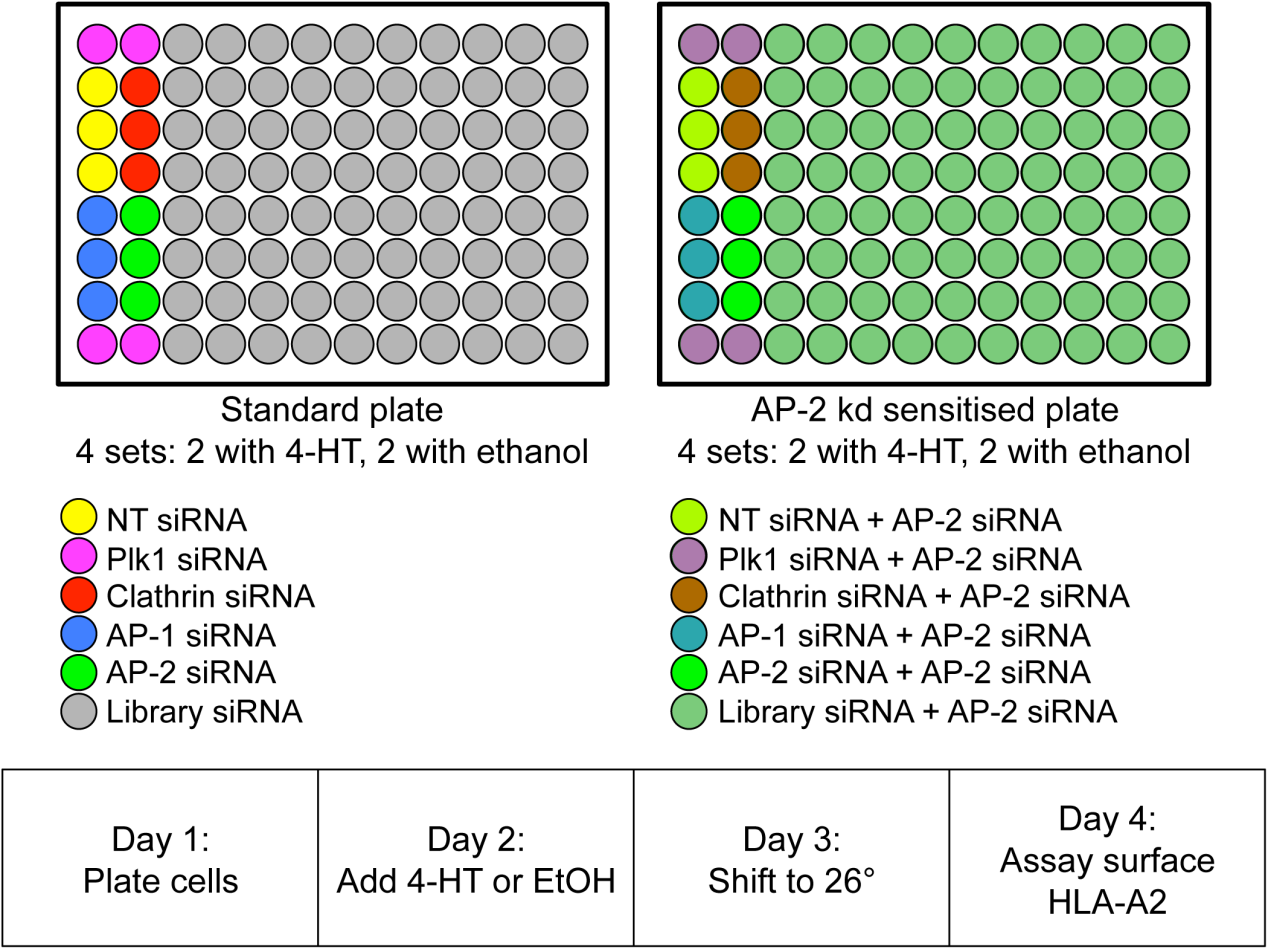
Designing the siRNA library screen. The screen was carried out on four sets of standard plates and four sets of plates that were “sensitised” by including siRNA targeting the AP-2 μ2 subunit in every well. The same positive and negative control siRNAs were added to the first two columns of every plate. Cells were added to the 8 sets of plates on Day 1; then on Day 2, half of the plates (2 standard and 2 sensitised) were treated with 4-HT and the other half with ethanol as a control. On Day 3, the plates were shifted to a lower temperature to enhance Nef activity, and on Day 4 the assays were carried out.

For our genome-wide screen, we used a Dharmacon library of 21,121 SMARTpool siGENOME siRNAs, arrayed in 267 96-well plates. The first 16 wells of every plate contained both positive controls (siRNAs targeting clathrin, AP-1, and AP-2) and negative controls (an siRNA targeting Plk1 and a non-targeting pool (NTPool)), while the remaining 80 wells contained library siRNAs (Fig 2). Eight copies of each plate were made, and an siRNA targeting AP-2 was added to every well in half of the plates, to “sensitise” the cells to Nef, because downregulation of MHC-I is more efficient in AP-2-depleted cells [7]. Next, we added HeLa cells stably expressing HLA-A2 and Nef-ER to the wells, thus introducing the siRNAs by reverse transfection. After 24 h, 100 mM 4-HT was added to half of the plates, while the other half were treated with the same volume of ethanol. After 48 h, the plates were shifted to 26°C, and after 72 h the cells were fixed, labelled for surface HLA-A2 using an Alexa Fluor 488-conjugated secondary antibody, and stained with the DNA dye Hoechst to provide an indication of cell number. A plate reader was used to measure the Alexa Fluor 488 and Hoechst fluorescence in each well. The experimental procedure is summarised in Fig 2.

**Fig 3:**
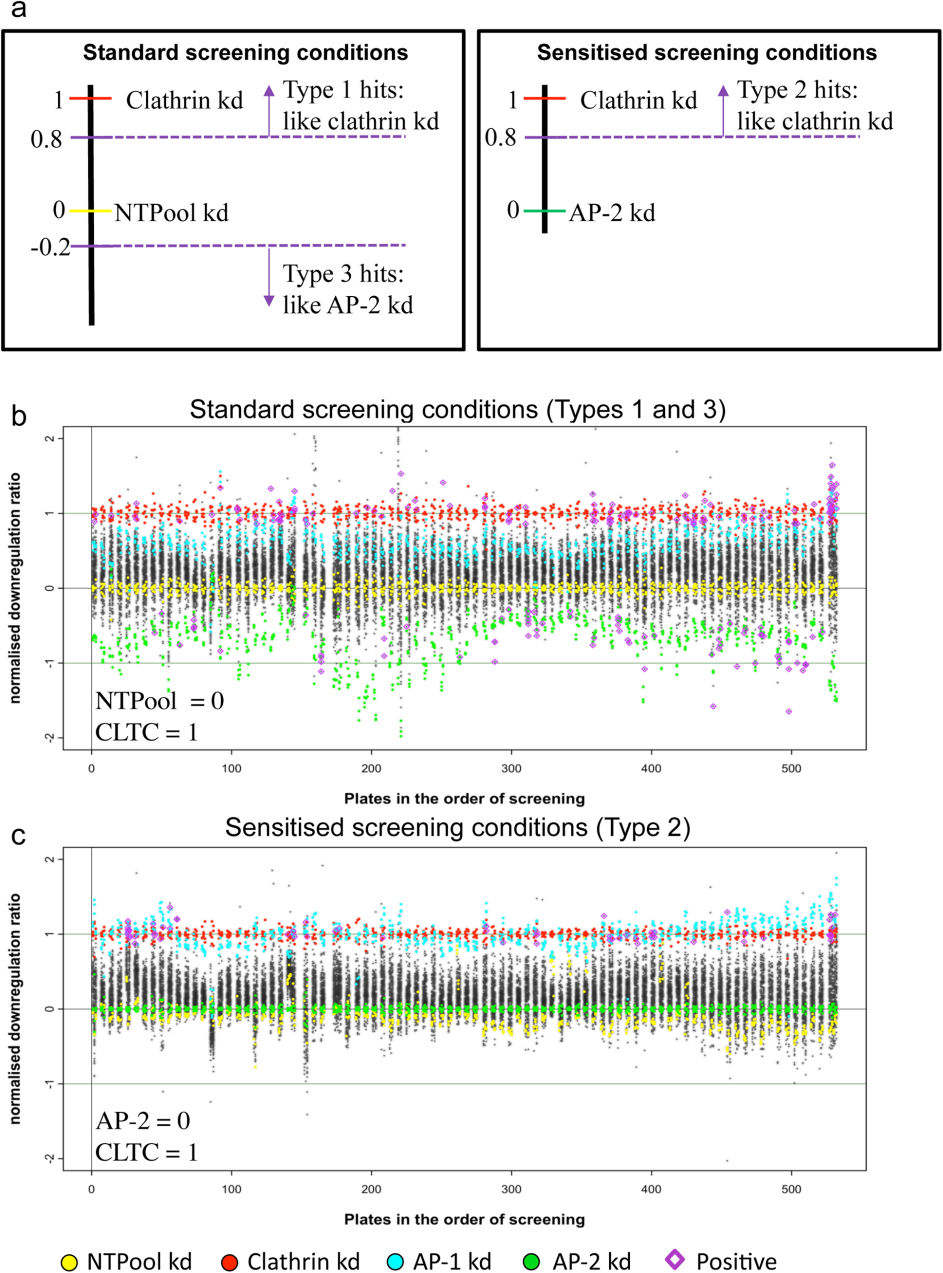
Results of the genome-wide screen, plotted according to downregulation ratio. a, Schematic diagram of the types of hits we were looking for and the criteria for each in terms of downregulation ratio. b, Normalised downregulation ratio under standard conditions, with the genes presented in order of screening. Selected positives (Type 1 and Type 3) are marked in purple. c, Normalised downregulation ratio under sensitised conditions, with the genes presented in order of screening. Selected positives (Type 2) are marked in purple.

In order to compare the amount of MHC-I downregulation between plates, a phenotypic metric was derived. We defined the MHC-I downregulation ratio as surface HLA-A2 in 4-HT-treated cells divided by surface HLA-A2 in ethanol-treated cells, after normalisation to both positive and negative controls. We used the clathrin knockdown as a positive control for both standard plates and sensitised plates, setting the value to 1, in order to make other knockdowns with a similar effect easier to spot. For the standard plates, used the NTPool as a negative control, setting the value to 0; and for the sensitised plates, used the AP-2 knockdown as a negative control, again setting the value to 0 (because AP-2 was knocked down in all of the wells, there was no difference in phenotype in the wells containing additional AP-2 siRNA, and these data were more reproducible than the NTPool data) (Fig 3A).

We then used these values to define three types of hits. First, in the screen carried out using standard plates (i.e., without adding AP-2 siRNA), knockdowns with a similar phenotype to the clathrin knockdown, defined as those with values of 0.8 or above, were classified as Type 1 hits. Second, in the sensitised plates, knockdowns producing a similar phenotype to the clathrin knockdown, defined as those with values of 0.8 or above, were classified as Type 2 hits. Third, in the standard plates, knockdowns producing a similar phenotype to the AP-2 knockdown, defined as those with values of -0.2 or below, were classified as Type 3 hits. This scheme is illustrated in Fig 3A.

The results are presented in Fig 3B for the standard plates and in Fig 3C for the sensitised plates, in order of screening. An interesting observations is that the AP-1 RNA, which produced only a moderate phenotype in the standard screen, produced a much stronger phenotype in the sensitised screen (i.e., in the presence of an AP-2 knockdown), virtually identical to the phenotype of the clathrin knockdown.

### Calculating the SSMD

**Fig 4:**
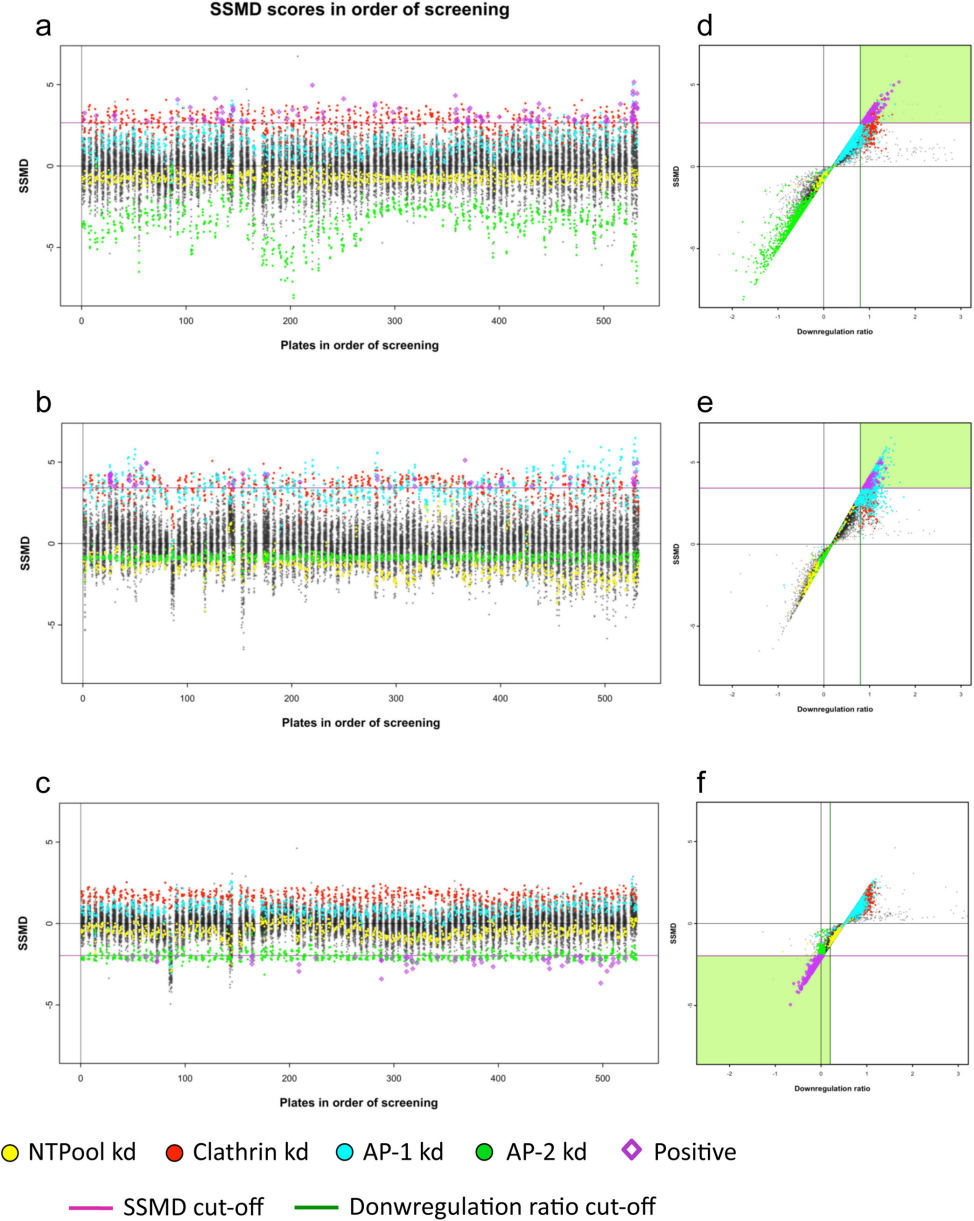
Results of the genome-wide screen, plotted according to SSMD. The SSMDs have different values from the downregulation ratios. Selected positives are marked in purple. In a-c, genes are presented in order of screening. a, Type 1 hits, selected from standard plates. b, Type 2 hits, selected from sensitised plates. c, Type 3 hits, selected from standardised plates. d-f, Downregulation ratio (x axis) plotted against SSMD score (y axis), using data from a, b, and c, respectively. The green shading marks the area from which hits were selected.

Next we calculated the strictly standardised mean difference (SSMD) for each SMARTpool. The SSMD is a measure of the strength of phenotype of a particular knockdown, and is also closely linked to how reproducible the phenotype is between the two replicate plates (i.e., the greater the standard deviation between the two replicates, the lower the SSMD for that particular knockdown). As a reference population for calculating phenotype strength, we decided to use the majority of the library knockdowns, apart from those with downregulation ratios in the top and bottom 5%. Fig 4A–4C, shows the SSMDs for each knockdown in order of screening, which were used to identify the three types of hits (1, 2, and 3, respectively). The cut-off values were selected based on the median SSMD for a particular positive control (clathrin for selecting Type 1 and Type 2 positives, and AP-2 for selecting Type 3 positives).

To illustrate how the SSMD modifies the ranking of SMARTpools when compared with the normalised MHC-I downregulation ratio, the two parameters were plotted against each other in Fig 4D–4F, with each plot next to the corresponding plot of the SSMD alone. For the majority of the library’s SMARTpools, there is a linear relationship between the downregulation ratio and SSMD score. However, there is also a small group of siRNAs that have a high downregulation ratio, but a relatively low SSMD score, and these are the SMARTpools that had low reproducibility between the replicate plates.

Other criteria that were used to formulate the hit list included a viability threshold, a standard deviation of <0.2 for two the technical replicates, and a lack of effect on surface MHC-I in the absence of 4-HT (see Materials and Methods for further details). The final hit list was cross-checked against three additional datasets: a HeLa cell microarray dataset [14], a HeLa cell proteome dataset (provided by Matthias Mann’s lab), and an siRNA targeting dataset (provided by Michael Boutros’s lab). The microarray and proteome datasets were used to ensure that the gene targeted by a particular siRNA was actually expressed in HeLa cells, and any siRNAs that targeted non-expressed genes were discarded bacause they were assumed to be off-target. The siRNA targeting dataset identified those siRNAs that targeted either no genes or more than one gene, and these were also discarded. In addition, siRNAs targeting genes that were used as positive controls in our screen (clathrin, AP-1, and AP-2) were not pursued, although they were useful indicators of how well the screen had worked. The full list of hits from the primary screen can be found in S1 File, and the raw data from both primary and secondary screens can be found at http://www.bioinformatics.cimr.cam.ac.uk/cgi-bin/mchoma_screen/mchoma_screen.cgi

### Validating the hits

**Fig 5:**
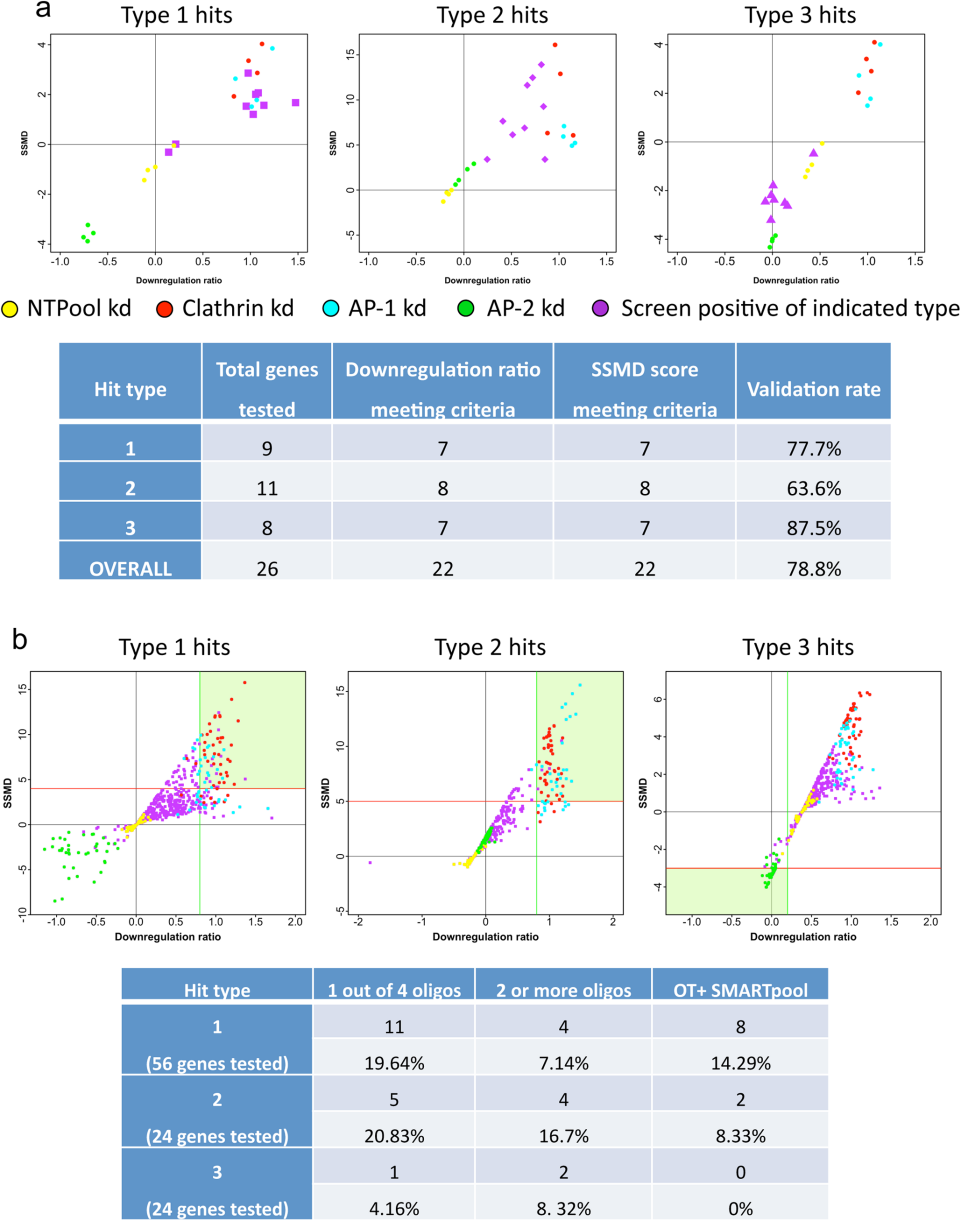
Results of validation experiments. a, Pilot experiment carried out using the same siRNA reagents as the primary screen. The downregulation ratio (x axis) is plotted against the SSMD score (y axis) for each type of hit. Most of the hits were validated. b, Validation experiment using different siRNA reagents. The downregulation ratio (x axis) is plotted against the SSMD score (y axis) for each type of hit. The green shading marks the area from which hits were selected. Very few of the hits came through when we used different reagents.

To validate our hits, we began by carrying out a pilot experiment in which the same reagents and assay were used on selected genes, to assess the reproducibility of the phenotypes observed in the primary screen. 26 siRNAs belonging to each of the three categories of hits were picked from the original library, together with 24 control siRNAs that had no effect in the screen. The results are presented in Fig 5A. Most of the knockdown phenotypes were reproducible, with an overall validation rate of 78.8%, and none of the negative controls affected Nef activity.

Although the primary screen and pilot validation screens used siGENOME siRNA reagents, for the full-scale validation we decide to use ON-TARGETplus (OT+) siRNA reagents, which were designed with an updated algorithm and chemically modified to limit off-target effects. We tested each of the four individual ON-TARGETplus siRNAs targeting a particular gene, as well as the four pooled together. Thus, for every hit from the primary screen, we obtained five new data points. A total of 100 genes were selected: 56 Type 1, 24 Type 2, and 24 Type 3, with four genes being classed as both Type 1 and Type 2. The results of the validation screen are presented in Fig 5B, showing all five of the data points for every gene. It is immediately apparent that that the reproducibility was much poorer than in the pilot validation screen, when we used the same reagents. Indeed, very few primary hits were validated.

Out of the hits that fell into the “verified” category (as marked by the green boxes in Fig 5B), ten in total were validated by the OT+ SMARTpool, and ten in total were validated by more than one oligo out of the OT+ SMARTpool. Out of these, only four hits were validated by both approaches. Most of the other hits validated by the OT+ SMARTpool had only one individual oligo producing sufficiently high scores to meet the validation criteria. However, in all but one case the single oligo had a different sequence from each of the four oligos in the siGENOME library (the one exception was FITM2, GeneID 128486). There were also two instances of a gene validated by multiple individual oligos, where one of the oligos shared its sequence with one of the oligos in the siGENOME set but the other(s) were different. In addition, three of the hits validated by a single oligo had a borderline second oligo, suggesting that they should not be ignored. Thus, in summary, out of the 100 genes selected for further analysis, 16 genes were validated by the two different approaches, but only 4 genes by >1 individual OT+ oligo and and also by the OT+ SMARTpool.

**Fig 6:**
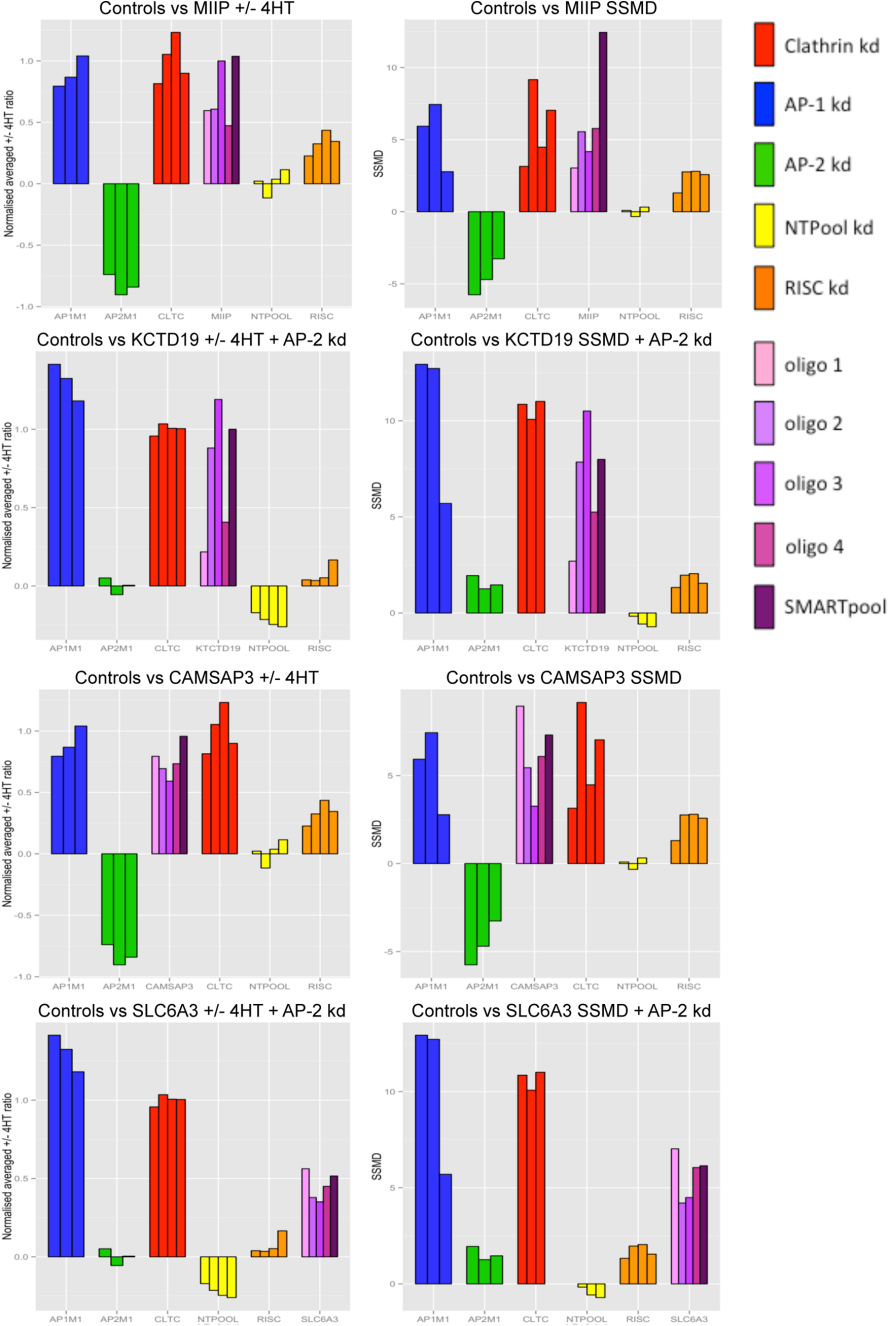
Detailed results of hits that validated with more than one siRNA, showing both downregulation ratios and SSMD scores. The scores for controls on the same plate as the genes of interest are also shown for reference.

These four hits validated by both approaches go by the gene names MIIP, KCTD19, CAMSAP3, and SLC6A3. Their knockdown phenotypes compared with positive and negative controls are shown in Fig 6. All of these genes passed very stringent criteria, and thus it is highly likely that they contribute to Nef-induced MHC-I downregulation. Further investigations should help to establish what these proteins actually do, and how their depletion affects Nef activity.

## Discussion

We have designed and carried out a genome-wide siRNA library screen to identify novel host factors that contribute to Nef’s ability to downregulate MHC-I. The system we used differs from a “normal” HIV infection, where the host cells are mainly CD4-positive T lymphocytes and the Nef is wild-type. Our screen was carried out on a modified HeLa cell line transduced with a Nef chimera. However, because the aim of the screen was to investigate the virus’s ability to evade the immune system and not to infect, we believe that our setup was optimal. HeLa cells are much easier to transfect with siRNA than T cells; they grow rapidly; and they are adherent, which makes them easy to handle and label. The finding that knocking down AP-1 inhibits Nef’s ability to downregulate MHC-I in both HeLa cells and T cells indicates that the underlying molecular mechanism is the same. One difference between HeLa cells and T cells is that nascent MHC-I is trafficked much more rapidly in HeLa cells. However, by incubating the HeLa cells at 26°C, MHC-I trafficking is slowed down, and this is thought to give Nef more time to capture the MHC-I and keep it off the cell surface [7,17]. Thus, although it will be important to validate our hits in HIV-1-infected T cells, our screen provides a solid foundation for future experiments.

To circumvent the adverse effects of high Nef expression on cell viability, we used an inducible form of Nef conjugated to the hormone-binding domain of the estrogen receptor. This allowed us to generate a stable cell line, ensuring reproducibility between experiments. Precisely why the Nef-ER chimera is inactive unless cells are treated with 4-HT is unknown, but our immunofluorescence results suggest that the 4-HT enables the chimera to be recruited onto membranes. The exact mechanism of action would probably be best addressed by a crystallographic study of Nef-ER in the active and inactive form. Even using an inducible system, we were never able to get more than moderate levels of Nef expression, and thus under standard conditions surface MHC-I typically dropped to 60–70% of control levels when we added 4-HT. In contrast, when HeLa cells are transiently transfected with Nef, surface MHC-I can go down to <10% of control in the highest expressors [7]. The relatively narrow dynamic range of our assay was one of the reasons we screened duplicate plates under sensitised conditions, where surface MHC-I expression was reduced to <50% control levels.

One of the most challenging aspects of carrying out an RNAi screen is determining how to analyse the data, because the analysis has to be tailored to the particular research question and experimental set-up. Working under the assumption that the vast majority of the siRNAs in the screen would have no effect on the phenotype of interest, we used the sample wells themselves as a negative reference for scoring hits, removing only the top and bottom 5% of the siRNAs as ranked by their downregulation ratio. This provides a more accurate estimate of the negative population than the negative controls, simply because there are many more experimental wells than negative control wells [19]. The same approach would not be appropriate for analysis of the secondary screen, because in this case most of the knockdowns were presumed to have an effect; thus, we used the two negative controls (NTPool and RISC-free) as our reference points. We chose SSMD as our screening metric for several reasons: it was designed and developed with siRNA screens in mind; it has a solid statistical basis; and it introduces an element of quality control [20].

The full list of hits from our primary screen can be found in S1 File. These hits were divided into three categories: those that inhibited downregulation under standard conditions (Type 1), those that inhibited downregulation under sensitised conditions (Type 2), and those that enhanced downregulation (Type 3). Out of 239 hits identified in the primary screen, 69 were discarded because of problems with the siRNAs themselves. A further 37 were discarded on the basis of not being expressed in HeLa cells. Out of the remaining 133 hits, 100 were selected for further analysis. In contrast to our previous screen for genes involved in clathrin-mediated endocytosis, we found no functional clusters of hits; they all belonged to different gene families, and many were of unknown function. We believe this highlights the value of unbiased screening, because it can direct the attention of further research towards previously unknown genes.

One surprising finding was that there was not more overlap between Type 1 and Type 2 hits. We had hoped that the sensitisation condition would enable us to find knockdowns in the same category as AP-1, which have only a moderate effect on MHC-I downregulation under standard conditions, but which have a much stronger effect when AP-2 is depleted; thus, we expected that any Type 1 hits would automatically also qualify as Type 2 hits. However, only four genes were found in both categories, and none of these were actually validated. One possible explanation might be that knocking down AP-2 not only sensitises the assay, it also leads to other effects brought on by the loss of a major player in endocytosis: e.g., it might trigger a different mechanism of downregulation.

The pilot validation experiment, which was performed using the same reagents as in the original screen, produced a validation rate of 79%. However, the larger validation screen, which used OT+ siRNAs with mainly different sequences, produced a validation rate of at best 16%, suggesting that although the data from the original screen were reproducible, many of the phenotypes were in fact off-target. This is a general problem when carrying out large-scale screens using RNAi reagents, which is not always taken into account. For instance, in a multiparametric genome-wide screen for modulators of endocytosis [21], assays carried out using the same siRNAs (remove comma) had a Pearson’s coefficient of 0.6±0.02, while the same assays carried out using six different siRNAs purported to target the same gene had a Pearson’s coefficient of only 0.047±0.01. In our own recent screen for endocytosis machinery and regulators, one of the most robust hits was from a SMARTpool knockdown of a gene subsequently found not to exist (LOC149478). When we looked for changes in mRNA expression levels in cells treated with this SMARTpool, using DNA microarrays, we found a strong reduction in expression of AP2M1, which explained why the phenotype was so strong (Nicola Hodson and MSR, unpublished observations).

Nevertheless, under the highly stringent conditions of the present study, we identified four hits that were validated by at least 2 individual oligos, as well as by the OT+ SMARTpool: two Type 1 hits, MIIP (GeneID 60672) and CAMSAP3 (GeneID 57662), and two Type 2 hits, SLC6A3 (GeneID 6531) and KCTD19 (GeneID 146212). Of the two Type 1 hits, MIIP (migration and invasion inhibitory protein) was initially identified in a yeast-two-hybrid screen as a binding partner for the insulin-like growth factor binding protein 2 (IGFBP2) and shown to inhibit glioma cell invasion [22]. Subsequent work demonstrated that MIIP can also interact with Cdc20, and that it may help to regulate mitotic progression [23]. CAMSAP3 belongs to the **ca**l**m**odulin regulated **s**pectrin-**a**ssociated **p**rotein family (member 3) [24], and contains a C-terminal microtubule-binding CKK domain. It has been suggested that CAMSAP3 and a closely related protein, CAMSAP2, maintain non-centrosomal microtubules and ensure correct organelle assembly and distribution [25].

Of the two Type 2 hits, the SLC6A3 gene encodes a dopamine receptor, also known as DAT, which has been studied mostly in the context of addiction and Parkinson’s disease [26]. KCTD19 is a relatively unknown gene with only a provisional status in the RefSeq database, which encodes a 926-residue protein. Its full name is potassium channel tetramerisation (KCT) domain-containing protein 19, because it contains two KCT domains. These domains, which are more commonly called BTB/POZ domains, are involved in protein-protein interactions. They are found in a number of proteins, several of which can bind to cullin 3 and participate in protein ubiquitination [27].

Although it is difficult to imagine a link between some of these hits and Nef, the whole point of a genome-wide screen is to uncover genetic interactions in unsuspected places. From a trafficking point of view, CAMSAP3 looks promising because of its interactions with microtubules, while the potential role of KCTD19 in ubiquitination makes it another interesting hit to follow up.

When compared with other genome-wide siRNA screens to find host genes involved in HIV infection and/or replication, the criteria set for our screen appear quite strict. Brass et al. [11]used the same siRNA library that we used, and defined the hits in their primary screen as those showing a difference of greater than or equal to 2 standard deviations from a given plate’s mean. For their secondary screen, they de-convoluted their original SMARTpool, and called any gene with at least one oligo replicating the original phenotype a validated hit, yielding a final list of 284 hits. Their high validation rate of 71% is not surprising given that they used the same reagents, and in fact it is similar to the validation rate we achieved in the present study using the same oligos. Another screen, carried out by König et al. [13], also set low criteria initially, but then the hits from the primary screen were scored on the basis of expression data, protein interactomes, and the NCBI HIV-1 Protein Interaction database, resulting in a final list of 295 hits. In a third screen, by Zhou et al. [12], SSMD was used to find hits in the primary screen, but the cut-off was set at 2; then the validation screen was carried out using a different set of SMARTpools rather than individual oligos. Their final list was 232 hits. In contrast, we ended up with just 4 high-confidence hits. This low validation rate is not entirely surprising: in our previous screen for genes involved in clathrin-mediated endocytosis, we ended up with only a handful of high-confidence hits, and these were all either known components of the endocytic machinery or subunits of the vacuolar ATPase. Thus, as mentioned above, we suspect that many of the reported hits from genome-wide RNAi screens are false positives due to off-target effects.

False negatives are another complication of RNAi screens, and we suspect that there are a number of genes in our screen that didn’t qualify as hits because they are just below the cutoff, which deserve further consideration. These include several known components of the CCV machinery. For instance, in our primary screen, ARF1 and GAK narrowly missed being scored as Type 1 and Type 2 hits, respectively; while AP2M1 was not quite strong enough to be picked up as a Type 3 hit, even though AP2M1 was used as a positive control on all of the plates. (moved to previous paragraph) Thus, our very stringent conditions may mean that some genes encoding proteins that contribute to Nef-induced downregulation of MHC-I did not make it into our final list. However, the availability of our dataset as an online resource means that it can be continually consulted, and may help to identify additional players in HIV pathogenesis.

## Materials and Methods

### Reagents, plasmids, and antibodies

4-Hydroxytamoxifen (Sigma), referred to as 4-HT, was prepared in absolute ethanol to a stock concentration of 129 mM and used at the specified final concentrations. Hoechst stain (Invitrogen) was prepared in ultrapure water to a stock concentration of 10 mg/ml, and used at a dilution of 1:1,000 for plate reader experiments, and 1:10,000 for immunofluorescence experiments.

pNef-ER was obtained through the NIH AIDS Research and Reference Reagent Program, Division of AIDS, NIAID, NIH (catalog no. 6454, contributed by S. Walk, K. Ravichandran and D. Rekosh). The pQCXIN vector was a kind gift from Andrew Peden (University of Sheffield). Both pNef-ER and pQCXIN were digested with EcoRI and BamHI, gel purified, and ligated to make the inducible Nef expression construct, which was then sequence verified.

The mouse monoclonal antibody against Nef, MATG020, was kind gift from Philippe Benaroch (Institut Curie, Paris). The mouse monoclonal antibody against HLA-A2, BB7.2, was purchased from BD Biosciences. The secondary antibodies, labelled with Alexa Fluor 647 and Alexa Fluor 488, were purchased from Invitrogen.

### Cell culture

HeLa M cells [28] were used throughout the study as a control cell line. The HLA-A2-expressing cell line was generated from HeLa M cells by Hewitt et al. [29], and was a kind gift from Paul Lehner (CIMR). The pQCXIN construct containing Nef-ER (described above) was transfected into Phoenix packaging cells (Orbigen, USA), and the resulting virions were used to infect the HLA-A2-expressing cells, generating a stable cell line expressing Nef-ER, called HeLa A2/Nef-ER. Because pQCXIN encodes a G418-resistant selectable marker, the HeLa A2/Nef-ER cells were cultured in medium containing a final concentration of 0.5 mg/ml G418, as well 1μg of puromycin/ml to maintain HLA-A2 expression.

The HeLa A2/Nef-ER cells were expanded in culture to generate 40 vials of cells, which were deposited in a liquid nitrogen cell bank on two occasions within a week of each other, to ensure the availability of cells with the same passage number throughout the screen. During the screen, two vials of cells were thawed and cultured for a week before they were used for transfections. Each batch was discarded after approximately 3 weeks in culture and a fresh batch was used.

For immunofluorescence microscopy, the cells were fixed in 3% v/v paraformaldehyde, quenched with 15 mM glycine, and permeabilised with 0.1% v/v Triton X-100 in PBS with 0.5% BSA. After hour-long incubations with primary and secondary antibodies, the samples were preserved using ProLong anti-fade reagent (Molecular Probes, Invitrogen). Images were acquired using a Zeiss Axioplan Fluorescence microscope with a 63x lens or an Improvision OpenLab Deconvolution inverted microscope.

### Screening and validation assays

A Matrix WellMate 8-channel microplate dispenser (Thermo Scientific) and a BiomekNX Laboratory Automation Workstation with a 96-channel head (Beckman Coulter) were used for 96-well plate liquid handling. A custom-made robotic enclosure with airflow technology and a UV lamp was built to provide a sterile work zone (BigNeat Containment Technology, Hampshire). Tissue culture-treated 96-well plates with clear bottom and black wells (Viewplates, Cat. No. 6005182, PerkinElmer) were used for the assay.

All siRNAs used in this study were produced by Dharmacon (Thermo Fisher Scientific). The genome-wide library contains 21,121 siGENOME SMARTpools arrayed in 267 96-well plates and was based on based on the human transcript RefSeq database V5.0-V8.0. The master plates contained 10 μl of 10μM siRNA in each well. The final concentration of the siRNA per well was 17 nM (2 pmol). For the sensitised part of the screen, an identical set of master plates was used with the addition of 2 pmol per well of ON-TARGETplus siRNA against AP-2 subunit μ2.

Reverse siRNA transfection was performed by pre-mixing Oligofectamine with Optimem (both from Life Technologies, Thermo Fisher) in a 1:8 ratio for 8 min to a total volume of 18 μl per well. Optimem was then added to a total volume of 100 μl, and the mixture was incubated for 20 min. This “mastermix” was then divided over 4 assay plates, 20 μl each, followed by the addition of 6,000 HeLa A2/Nef-ER cells per well. The plates were then gently vortexed for ~10 sec and placed in the 37°C incubator without stacking. 24 h after transfection, the growth medium was changed from standard to G418-containing medium, with the addition of 4HT to a final concentration of 100 nM or an equal volume of absolute ethanol, using the Matrix WellMate 8-channel microplate dispenser under sterile conditions. 48 h after transfection and 24 h after the change of growth medium, the plates were moved into an incubator kept at 26°C. 72 h post-transfection and 48 h post-addition of medium with 4HT to half of the plates, the cells were fixed and stained for surface MHC-I, together with Hoechst as an indicator of the number of cells per well. A 2103 Wallace EnVision multilabel plate reader (Perkin-Elmer Life Sciences) was used to read the fluorescent signal of the Alexa Fluor 488 (surface MHC) and Hoechst from each plate, using appropriate optical filters and a bottom-reading mode. In each well, 52 points spread on an 8x8 round grid, spaced by 0.79 mm, were scanned. Usually, 24 plates were scanned as a batch.

### Data analysis

MySQL (client version 5.1.63), a relational database management system, was used to store data associated with the RNAi screening project on a local server. A web-based front-end phpMyAdmin (version 3.3.2) was set up to manage the database, build database structure and work with the data. The data were assembled using Perl scripts and imported into MySQL by Nikol Simecek (CIMR). The database can be accessed at: http://www.bioinformatics.cimr.cam.ac.uk/cgi-bin/mchoma_screen/mchoma_screen.cgi

The database was built using the gene symbols provided in the Dharmacon library in 2005, and many gene symbols have been changed since then. It is therefore advisable to use Gene_ID when searching for a gene of interest.

Data analysis was performed in R. The data were scaled and underwent plate-wise background correction and normalization within each fluorescent channel. For each treatment, the Alexa Fluor 488 (surface MHC-I) reading was divided by the Hoechst (DNA) reading for each well, to determine the amount of surface MHC-I per cell. This value (surface MHC-I/DNA ratio) was then used to calculate average and standard deviation between a pair of technical replicates. The downregulation ratio was calculated as the surface MHC-I/DNA ratio in the presence of 4HT (average of duplicates), divided by the surface MHC-I/DNA ratio in the absence of 4HT (average of duplicates). The ratio’s error was also calculated following a formula for error propagation for division of two means. Downregulation ratio was normalized to controls. SSMD was used to calculate the power of the screen and the individual controls.

There were several rounds of quality control for the data at each stage. The list of positive genes was generated using the criteria summarized below:

- Cell viability. The DNA reading had to be 0.2 or above.
- “Interesting” MHC normalized ratio, and small SD (<0.2 of the ratio). An interesting ratio was defined as between 0.8 and 5 for Type 1 and Type 2 positives, and between -5 and 0.2 for Type 3 positives.
- No major effect on MHC surface levels under -4-HT conditions
- SSMD was then used for ranking the positives, using a cutoff based on the median of the positive controls’ SSMD value for each type of hit.
- Microarray expression data. Genes shown not to be expressed were excluded unless they were found in the HeLa proteome dataset.
- Proteome expression data. Genes not found in this dataset were excluded unless they were found to be expressed in the microarray dataset.
- siRNA target information. All siRNAs predicted to target either no genes or more than one gene were excluded.

### Validation

The data were processed in much the same manner as for the primary screen and the pilot validation study. Data points were corrected for background using the PLK1 knockdown, normalised to the median of each plate in each channel measured, and after calculating the downregulation ratio separately for each of the biological replicates, the values were normalised to the controls on each plate, with clathrin always set as 1 and NTPool set as 0 under normal conditions and AP-2 set to 0 under sensitised conditions.

The criteria used for hit selection in the pilot validation experiment were a combination of downregulation ratio and SSMD score. The requirement for Type 1 hits was a downregulation ratio of over 0.8 and an SSMD score of 2.1; for Type 2 hits, the requirement was a downregulation ratio of over 0.5 and an SSMD score of over 5.1; and for Type 3 hits (i.e., hits increasing downregulation), the requirement was a downregulation ratio of less than 0.2 and an SSMD score of less than -2.

For the full-scale validation screen, the downregulation rate cut-off for validated hits was slightly lowered, while the SSMD score cut-off was adjusted to take into account the data trends observed in the validation experiment. The cut-offs used were SSMD >4 and downregulation ratio >0.6 for Type 1 hits; SSMD >5 and downregulation ratio > 0.4 for Type 2 hits; and SSMD < -2 and downregulation ratio < 0.2 for Type 3 hits.

## Supporting Information

S1 File Full list of hits from the primary screen. (XLSX)
